# Rasch analysis of the modified version of the Postural Assessment Scale for Stroke patients: Postural Stroke Study in Gothenburg (POSTGOT)

**DOI:** 10.1186/1471-2377-14-134

**Published:** 2014-06-19

**Authors:** Carina U Persson, Katharina S Sunnerhagen, Åsa Lundgren-Nilsson

**Affiliations:** 1 Institute of Neuroscience and Physiology, Rehabilitation Medicine, Sahlgrenska Academy, University of Gothenburg, Per Dubbsgatan 14, Gothenburg S-413 45, Sweden

**Keywords:** Assessment, Outcomes, Reliability, Validity, Rehabilitation, Rehabilitation medicine, Rasch model

## Abstract

**Background:**

The modified version of the Postural Assessment Scale for Stroke Patients (SwePASS) is a new ordinal outcome measurement designed to assess postural control in patients with stroke. Before implementation of SwePASS into the clinical setting, it is necessary to know its measurement properties. Thus, the aim of the study was to evaluate the measurement properties of the SwePASS.

**Methods:**

Rasch analysis, based on data of 150 SwePASS assessments was made the first week after stroke onset. The measurement properties referred to were unidimensionality, local independence, invariance, category function, targeting of persons and items and the reliability.

**Results:**

The initial analysis showed disordered thresholds in four items. After adjustment of the scoring categories, this was resolved. However, analyses of local dependency revealed correlations between two of the items. These two items were collapsed into one. After adjustments, the person separation index that acts as an indicator of the whole model fit was 0.96. The adjusted SwePASS is a global scale that works the same way regardless of gender, age and location of stroke lesion. Overall, the population had better postural control than was targeted with the items in the scale.

**Conclusions:**

Rasch analysis of the adjusted SwePASS showed that the scale was unidimensional. In SwePASS, equal capacity in postural control provides the same response to an individual item in patients with stroke, regardless of gender, age and location of stroke lesion. Regarding clinical implications, before introducing SwePASS in clinical routine and to confirm the results, further research including a larger sample with poorer postural control is suggested.

## Background

The Modified Version of the Postural Assessment Scale for Stroke Patients (SwePASS) [1,2] (see Additional file 1) is an ordinal scale aimed for use in stroke rehabilitation and research. The SwePASS is a modification of the original French version called the Postural Assessment Scale for Stroke Patients (PASS) [3] in terms of different definitions of categories. The PASS [3], a clinical tool that assesses lying, sitting and standing, for monitoring patient’s weekly progress, was developed by Benaim and colleagues [3], from the Fugl-Meyer Assessment of balance and mobility [4] and the BL Motor Assessment [5]. In the development of the PASS, poor postural performance was related to impairment in body stabilization [3] i.e. maintain a posture and ensure equilibrium. The construct derived from the theory for the PASS, described by Benaim et al. [3], involves terms of both postural control and postural performance. The items of the SwePASS may be linked to the categories related to activities of maintaining (d410) and changing (d415) a body position in lying, sitting and standing, as described in the mobility chapter (d4) of the International Classification of Function (ICF). Thus, to clarify the content validity, the construct that SwePASS refers to is postural control of persons with stroke, as the ability in body stabilization during every day activities of lying, sitting and standing.

The SwePASS has been shown to be a highly reliable [1] and valid [2] scale in acute stroke as well as to be responsive to change over time [6]. This suggests that the SwePASS may be a reliable and a usable clinical indicator of postural control in activities of lying, sitting and standing*.* Whether the SwePASS scale can be regarded as unidimensional, is not clear. Unidimensionality of the scale will support the assumption that one construct is assessed. Unidimensionality is required in order to sum the item scores into a valid total score. For the total score to be a valid indicator of the person’s ability (i.e. does it makes sense to report a single score for a patient’s performance on the SwePASS?) there must be evidence of the internal construct validity and reliability of the SwePASS. One way to provide evidence that the total sum score of the items holds adequate internal construct validity is to use Rasch analysis [7-10]. By using this method, one can also change a nonlinear transformation of ordinal raw scores into interval measures, assuming that fit to the Rasch model is supported. In addition, using Rasch analysis is also about testing hypotheses. The hypothesis is that the SwePASS is measuring one single construct, here postural control as the patient’s ability in body stabilization (that is, maintain a posture and ensure equilibrium) during every day activities of lying, sitting and standing. The hypothesis about the hierarchy of the items in the SwePASS concerns that the items in sitting and lying are the easiest ones and that the items in standing are the most difficult ones. The aim of the present study is to evaluate the construct validity of the SwePASS, more specifically to evaluate the following measurement properties: unidimensionality, targeting of persons and items, category function, local independence, invariance, and the reliability of the SwePASS using Rasch analysis [7].

## Methods

### Participants, setting and instrument

The inclusion criterion was a first-ever stroke, defined according to the World Health Organization [11]. Exclusion criteria were co-morbidities such as leg amputation, a diagnosis of dementia or severe psychiatric diseases. The Regional Ethics Committee of Göteborg, Sweden, approved the study protocol. Written informed consent was obtained from all participants or their next of kin prior to participation, in compliance with the ethical principles set forth in the Declaration of Helsinki [12].

Data were used of 150 patients with a first-ever clinical stroke, based on SwePASS assessments by physiotherapists. The data were collected at a stroke unit at Sahlgrenska University Hospital, Göteborg, Sweden between days 4 and 7 after stroke onset. The current study is part of the Postural Stroke Study in Gothenburg (POSTGOT).

The SwePASS, like the PASS, contains 12 items, based on a four-category scale from 0 to 3. A category of 0 implies that the patient cannot perform the activity and/or has impaired postural control. A category of 3 implies that the activity can be performed without any help or support or with the best possible performance regarding a specified support or time. Each item of the SwePASS has three thresholds (between the categories 0-1, 1-2 and 2-3), one less than the number of response categories. A threshold is the point at which the probability of a response in either one of two adjacent categories is equal. In items 4 and 7-9, the patient is required to maintain a position for a specific length of time. The remaining items are scored according to different levels of support. When the scores of each item are added, the sum score of SwePASS will be between 0 and 36, where a higher score indicates better postural control.

### Rasch analysis of SwePASS

Initially, to determine which of the two Rasch models was suitable, the Rating Scale Model or the Partial Credit Model [13], a Likelihood-Ratio test was performed in the RUMM2030 [14]. The Rating Scale Model indicates that a set of items share the same rating structure, while the Partial Credit Model specifies that each item has its own rating scale structure. The unidimensionality and invariance of the SwePASS were continuously tested during the analyses. This was done by evaluating the overall fit, the category function, individual fit of the items and persons (residuals), local independence, reliability, and the differential item functioning (DIF) of items. Finally, unidimensionality was further tested using the method described by Everett Smith [15].

To assess the overall summary statistics with standardized mean person and item fit, a chi square item-trait interaction statistic was performed [16]. A non-significant probability value (Bonferroni adjusted) and standardized fit residuals of persons and items between ±2.5 indicate adequate fit or a perfect fit, the overall item and person residuals should display a mean of 0 and a SD of ±1 [17,18]. The Person Separation Index (PSI) was used to assess reliability. This supplies an estimate of the internal consistency of the scale, where a minimum PSI value of 0.90 is required for individual use.

The ordering of the thresholds of the response options were observed and collapsed in case of disordering [19]. In a rating scale, the ordinal numbering deals with that each level is fundamentally defined to represent a higher level of functioning. Category disordering (reversed numbering of categories) occurs when the ordinal numbering of categories does not accord with their fundamental meaning.

There are two types of local independency of items [6,20]. First, there is response dependence, i.e. when the probability to affirm an item is influenced by the response to another item [19]. Response dependence was suggested from values above 0.3 of the residual correlations for each pair of items [17]. Response dependency is dealt with by combining the locally dependent items into a larger item, in a ‘testlet’. Second, there is trait dependency, which is related to multidimensionality [20]. To discover any possible multidimensionality, two subsets of items that differ the most, in terms of negatively and positively loadings on the first principal component analysis of residuals, are selected. This means, that person abilities and their standard error were computed separately for the items loading positively and those loading negatively on the first principal component. Each person’s ability (and SE) is then compared using a t-test. If less than 5% of all the t-tests are significant, then no significant difference between the items of the two dimensions can be expected. Accordingly, the scale is unidimensional [19]. On the assumption that the content of the scale is unidimensional, the estimate of person’s ability’ to body stabilization during everyday activities in lying, sitting and standing should be the same on any subsets of items from that scale [15].

Differential Item Functioning (DIF) [21] is a form of item bias that can occur when different groups in the sample give different responses to an individual item, despite equal levels of the underlying trait, i.e. capacity in postural control. DIF is evaluated by conducting an analysis of variance (ANOVA), in this study for gender, age group (Group 1 ≤ 76 years, group 2 ≥ 77 years) and stroke localization (left or right hemisphere).

Using an item info curve, targeting was also visually assessed by comparing the threshold distribution graph of persons and items on the same logit scale. With a good targeting, the mean ability and the mean item difficulty are both close to zero [19]. Details on the procedure of the Rasch analysis are available in several publications [10,19,22-24].

### Statistical software and sample size issues

Descriptive analysis of frequencies of ischemic or haemorrhagic stroke and median age, was carried out using the Statistical Package for Social Sciences (SPSS^©^) (Version 17 SPSS Inc., Chicago, IL, USA). The Rasch analysis was made using RUMM2030 software© [14]. A significance value of 0.05 was used throughout, and Bonferroni correction was applied to adjust for the multiple numbers of tests [17].

## Results

The median age of the total sample of 150 patients was 76 years (min 34 and max 95 years). There were 86 men (57%). One hundred thirty-eight of the patients (92%) had ischemic stroke, while the remaining 12 patients (8%) had haemorrhagic stroke. The SwePASS assessments were made at a median of 5 days after stroke onset.

According to the Likelihood-Ratio test, SwePASS did not meet the requirements of a rating scale model. Thus, the Partial Credit Model, a polytomous version of the Rasch analysis was used.

### Analysis 1

Initial fit of the scale to the Rasch model was good (Table 1, Analysis 1), with a non-significant chi-square value. Both the item fit and the person fit were acceptable and the reliability (PSI) was high. However, further analysis of the items revealed disordered thresholds for four of the items: Item 4, Item 7, Item 8 and Item 10. Consequently, the categories in these items were rescored and collapsed from four to three numerically. Thus, for Item 4 and Item 10 the response categories 0 and 1 were collapsed using a 0012 code. For Item 7 and Item 8 the response categories 1 and 2 were collapsed using a 0112 code. Moreover, since there is no practical difference, in terms of ability to body stabilization, between a person who has not that ability and one who requires the help of two persons, it was decided to merge the first two categories in Item 1, Item 2 and Item 3 into one, using a 0012 code.

**Table 1:** Fit of the modified version of Postural Assessment Scale for Stroke Patients (SwePASS) to the Rasch model

**Analysis**	**Item fit residual mean (SD)**	**Person fit residual mean (SD)**	**x**^**2**^	**DF**	***P***	**PSI**	**% tests**	**95% CI**
1. All items	-0.41 (0.66)	-0.16 (0.20)	34.78	24	0.07	0.97	3.33	-0.002-0.068
2. Rescoring	-0.33 (0.51)	-0.14 (0.18)	24.90	24	0.41	0.96	3.33	-0.002-0.068
3. Dealing with local dependency	-0.33 (0.55)	-0.14 (0.18)	22.79	22	0.41	0.96	2.5	0.025-0.095
Ideal values	0.00 (1.0)	0.00 (1.0)			>0.05*	>0.90	<5.00	Lower bound <0.05

SD; Standard Deviation, x^2^; Chi-square value, DF; Degrees of Freedom, P; P-value, PSI; Person Separation Index,% tests; percentage of positive t-test, 95% CI; 95% Confidence Interval, *; Bonferroni adjusted (Bonferroni correction p 0.004167 for all 12 items in analyses 1 and 2 and p 0.004545 for 10 items and the testlet in the analysis 3).

### Analysis 2

The analysis of the rescored items lead to a good overall fit to the Rasch model with a non-significant chi-square value, acceptable item and person fit residuals and no more than marginally reduced reliability and no remaining disorder of thresholds (Table 1). However, a local dependency was found between Items 6 ‘Standing with support’ and 11 ‘Sitting down from standing up’. Consequently, those items were combined into one, a testlet.

### Analysis 3

**Figure 1:**
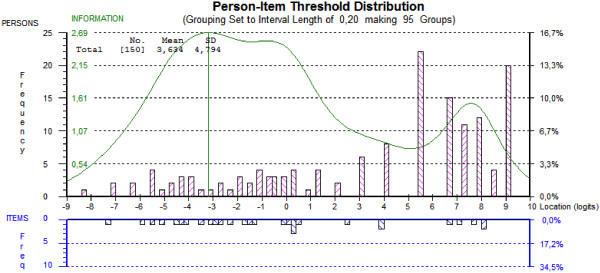
**Targeting of persons and items.** The figure illustrates the ability of the SwePASS to cover the range of the latent trait, postural control, in the study population. This is done by comparing the distribution of persons (upper plot) and items (lower plot) on the same logit scale, based on the final solution for SwePASS. Towards the right of the scale, postural control increases. Perfect matching happens when both the distributions of persons do not fall outside the distribution of items and when distributions have a mean of zero logits.

In the third and final analysis, using the testlet solution for local dependence, with an overall fit to the model (as shown in Table 1), as indicated by the average items and persons residuals within the -2.5 to 2.5 range and a non-significant Bonferroni-adjusted chi-square, the data was fully compatible with all assumptions of the model. The scale appeared highly reliable (PSI 0.96) and unidimensional with a percentage of significant t-test <5% (Table 1). Table 2 displays that all individual items and persons fitted the model, there were no further locally dependent items and there was no DIF. Table 2 also presents the item locations, the standard error and a rescoring key for the final solutions. Figure 1 illustrates the final solution with item info curve. The positive mean person ability value of 3.63 (SD 4.79) indicated that the sample as a whole had an average higher level of ability (here postural control) than the average difficulty of the items of the scale (centralized by default at 0 logits), thus indicating that the scale was not optimally targeted to the sample.

**Table 2:** Item location, item fit and scoring key for the final solution of the analyses

**Item**		**Location**	**Std Dev**	**Item fit residual mean**	**x**^**2**^	**DF**	***P***	**Max score**	**Rescored**	**Scoring key**			
1	Supine to affected side lateral	-3.61	0.32	-0.03	1.12	2	0.57	2	Yes	0	0	1	2
4	Sitting without support	-3.25	0.31	-0.14	0.18	2	0.92	2	Yes	0	0	1	2
12	Sitting on the edge of bed to supine	-3.23	0.27	-0.54	0.29	2	0.86	3		0	1	2	3
6 + 11	Standing with support & Sitting down from standing up	-2.30	0.17	-0.63	0.22	2	0.90	6		*	*	*	*
3	Supine to sitting up on edge of bed	-2.18	0.30	0.33	0.56	2	0.75	2	Yes	0	0	1	2
2	Supine to non-affected side lateral	-1.94	0.30	-0.26	0.99	2	0.61	2	Yes	0	0	1	2
5	Sitting to standing up	-1.89	0.25	-1.42	0.53	2	0.77	3		0	1	2	3
10	Standing, picking up a shoe from the floor	1.35	0.26	-1.11	0.72	2	0.70	2	Yes	0	1	1	2
7	Standing without support	2.10	0.26	0.29	8.94	2	0.01	2	Yes	0	1	1	2
8	Standing on non-paretic leg	7.33	0.20	-0.06	3.88	2	0.14	2	Yes	0	1	1	2
9	Standing on paretic leg	7.63	0.16	-0.07	5.36	2	0.07	3		0	1	2	3

Location; on the logit ruler from the easiest (-) to the most difficult (+) item, Std Dev; Standard deviation, x^2^; Chi-square, P;Chi-square probability (Bonferroni adjusted p 0.004545). *For each item = max score 6.

## Discussion

At this initial validation stage, the Rasch analysis provided evidence that SwePASS did not work as originally intended. On the basis of these initial findings, the scale was modified. After adjustments of categories and local dependencies, this first Rasch analysis of the SwePASS shows that the SwePASS could be considered as unidimensional and that it works as a global measure of postural control as the ability to body stabilization during every day activities at an individual level in patients with stroke, regardless of gender, age and location of stroke lesion.

At the same time, the initial analysis highlighted a number of potential problems. For instance, 4 of 12 items showed disordered thresholds. From a clinical perspective this is unsatisfactory, both for the patient and for the physiotherapist, who must be able to trust the results of the measurement instrument. Particularly, it is important for a physiotherapist to know that a scale has sound measurement properties based on items that reflect the hypothesized structure and hierarchy of the construct (a logical progression of the ability to body stabilization). An improvement in ability or a good ability shall give a higher or a high score, while deterioration in ability or a low ability must give lower or a low score. Using a scale with disordered thresholds, a change in categories will be difficult or even impossible to evaluate. A misjudge of a patient’s postural control may have consequences on the patient’s self-efficacy and to safety. Disordered thresholds, which may result from several different factors, are likely caused by the item designer as a failure of the hypothesis behind the items. Counter-intuitive, this was addressed by re-scoring according to statistical criteria as well as to clinical judgement. For instance, in Item 4 (‘Sitting without support’), raters may find it difficult to discriminate between the two first categories (0 = Cannot sit; 1 = Can sit with slight support, for example by 1 hand), as the word “slight” may be too vague despite the extended explanation (‘by one hand’), thus leading to disordered thresholds. The new created observational framework of 3 categories for this item may be discriminated more easily by the physiotherapist (Cannot sit or require support for sitting / Can sit without support >10 s / Can sit without support > 5 min). In Item 7 (‘Standing without support’), the category 3 does not seem to add much information of postural control. Furthermore, in Item 8, (‘Standing on the non-paretic leg’), the raters may find it difficult to between the narrow time ‘cut-offs’ in category 1 and 2. By contrast, Item 10 (‘Standing, picking up a shoe from the floor’), the judging does not include any time-restriction, but instead includes a factor related to need of support. Clearly, picking up a shoe from a standing position is a demanding task. If either the patient or the physiotherapist has doubts about the patient’s chance to perform the task, they may avoid trying, automatically resulting in a score of 0. Standing and picking up a shoe is both a complex and dual task, why being able to implement it, whether with one or two persons support, means more ability to body stabilization than not being able to do it at all.

In contrast with the Items 4, 7, 8 and 10, the rescoring of Items 1-3 was based only on clinical implications and not disordered thresholds. Theses 3 items (rolling on the sides and postural change from supine to sitting) were rescored, as we considered that for the purpose of measuring postural control in such relatively easy activities the differences between 0 (cannot perform the activity) and 1 (can perform the activity with the help of two persons) would be minimal. Indeed, after these modifications, the difficulty hierarchy of the items changed and became more consistent with the expected progression in capability of postural control.

The local dependency revealed in the SwePASS between Item 6 ‘Standing with support’ and Item 11 ‘Sitting down from standing up’ might be explained by the fact that the two items both test the ability to stand up. To work as intended, the scale should not include one or more similar items, both in terms of affection of estimation as well as of time efficiency and effort. As a consequence of the local dependency revealed in SwePASS, the items 6 and 11 were combined into a testlet. This solution led to a fit to the Rasch model, with only a marginal reduction of the reliability value, indicating that the SwePASS has the potential to be used at the individual level.

Since stroke patients represent a very heterogeneous group, the result demonstrating nonexistence of DIF is important and positive for the clinician. In SwePASS, the responses of persons to items are determined only by the patients’ ability of postural control and are not influenced by their gender, age or stroke location. Consequently, de facto facilitates the interpretation of the scale.

According to the item location, defined in logits and presented in Table 2, it is fully expected that the items associated with supine and sitting are less demanding on postural control than the items associated with standing positions. As expected the two items involving the procedure of standing on one leg turned out to be the most difficult ones. As the clinical experience suggest, it is also expected that the ‘Supine to affected side lateral’ (Item 1) is less difficult than ‘Supine to non-affected side lateral’ (Item 2).

Recently, La Porta and colleagues performed a Rasch analysis of the Berg Balance Scale (BBS) [25]. BBS is another clinical scale to assess balance in elderly [26,27], including patients with stroke, with several similar items as in the SwePASS. In that Rasch analysis, disordered thresholds were detected in 11 of 14 items [25]. Thus, compared to the current study of SwePASS, the problem with disordered thresholds was even larger in the BBS.

There are three major limitations of the study which need to be discussed, namely the suboptimal targeting, the sample size and the inability to provide a transformation table from raw scores to interval data. The results of this study show that the SwePASS is not optimally targeted to the examined population. There is a need in both clinic and research to assess even the patients that are most severely affected. This is done in clinic. However, the most severely ill patients were not included in the study. This was partly based on the view of the responsible physician that it might be unethical to ask for informed consent in a medical more or less critical condition or at least in a chaotic situation for the relatives. If these patients had been included, it is plausible that more patients had demonstrated a lower capacity in postural control, as shown at the lower end of the scale. The suboptimal targeting is not only a limitation of the study, but also a negative finding. The small number of data (participants) in this study (n = 150) and the poor targeting means no optimal power to detect and adjust for problems in the scale, only give indications of possible problems. Relative to the sample size, even if the data satisfy the Rasch model expectations, the raw score obtained from the scale could not be converted into an interval scale whose estimated unit of measurement is the logits. The transformation of ordinal data into interval data, leading to the possibility of using parametric statistical analyses, requires a larger population than was used in the current study. As a guiding principle, Linacre [28,29] has proposed a sample size of 250 for accurate estimation and appropriate degree of precision using Rasch analysis, regardless of targeting of persons to the scale. From a clinical point of view, interval data would simplify the interpretation of change in postural control using SwePASS, as well as allowing a presentation of the results in terms of a sum score. In addition, there is always a risk of type I or type II errors when analysing data with Rasch analysis and this risk becomes much higher if the sample is small and not well targeted, as in this study. However, even considering that some findings of the analysis are highly significant, further studies with larger samples are needed to confirm the findings. Before then, it cannot be advocated that that categories of the SwePASS should be changed.

A review on balance scales in 2009 [30] showed that there were around 30 instruments measuring balance and postural control, and this number is constantly increasing [31,32]. The SwePASS is specifically developed for persons with stroke, modified according to clinical relevance and has also been shown that be quick to perform in clinic [1]. SwePASS has been investigated in different ways with methods that are recommended to handle ordinal data, concerning its psychometric properties regarding previously presented results of reliability [1] and responsiveness [6] as well as the current internal construct validity. The psychometric evidence of the predictive validity [2] may be interpreted very cautiously. Even if the total score was used dichotomized, it can be considered bias before dealing with adjustments of scoring categories and local dependency. Overall, the new scale, the SwePASS, may be a scale of interest to both the clinician and the researcher.

## Conclusion

In conclusion, Rasch analysis of the adjusted SwePASS shows that SwePASS is unidimensional. SwePASS works as a global outcome measurement of capacity in postural control in patients with stroke. Before introducing SwePASS in clinical routine and to confirm the results, further research including a larger sample with poorer postural control is suggested.

## Abbreviations

DIF: Differential Item Functioning; PSI: Person Separation Index; SwePASS: The modified version of the Postural Assessment Scale for Stroke Patients.

## Competing interests

The authors declare that they have no competing interests.

## Authors’ contributions

CUP contributed to the study design, acquisition, analysis and interpretation of data, drafting and editing of the manuscript. KSS contributed the overall study design, acquisition, analysis and interpretation of data and manuscript editing. ÅLN contributed to the analysis and interpretation of data and manuscript editing. All authors’ have read and approved the final version submitted for publication.

## Pre-publication history

The pre-publication history for this paper can be accessed here:

http://www.biomedcentral.com/1471-2377/14/134/prepub

## Supplementary Material

Additional file 1 The modified version of PASS - SwePASS. Click here for file
