# Supplementary material for: Rasch analysis of the modified version of the Postural Assessment Scale for Stroke patients: Postural Stroke Study in Gothenburg (POSTGOT)
Source: BMC Neurol. 2014 Jun 19;14:134. doi: 10.1186/1471-2377-14-134 (PMC4069346; doi:10.1186/1471-2377-14-134)
Supplement: Additional file 1 — The modified version of PASS - SwePASS. [file 1471-2377-14-134-S1.pdf]

The modified version of PASS - SwePASS

Item	Scale	Item	Scale
**1. Supine to affected side lateral**		**7. Standing without support**	
Cannot perform the activity	0	Cannot stand without support	0
Can perform the activity with support from 2 persons	1	Can stand without support for 10 s or leans heavily on 1 leg	1
Can perform the activity with support from 1 person	2	Can stand without support for 1 minute or stands slightly asymmetrically	2
Can perform the activity without any help	3	Can stand symmetrically without support for more than 1 minute and at the same time draw hand/s from forehead to neck (like pulling your fingers through your hair) alternating with arm/s hanging parallel to the trunk to avoid tiredness	3
**2. Supine to non-affected side lateral**		**8. Standing on non-affected leg**	
Cannot perform the activity	0	Cannot stand on the non-paretic leg	0
Can perform the activity with support from 2 persons	1	Can stand on the non-paretic leg up to 5 seconds	1
Can perform the activity with support from 1 person	2	Can stand on the non-paretic leg for more than 5 seconds	2
Can perform the activity without any help	3	Can stand on the non-paretic leg for more than 10 seconds	3
**3. Supine to sitting up on edge of bed towards the non-affected side**		**9. Standing on affected leg**	
Cannot perform the activity	0	Cannot stand on the paretic leg	0
Can perform the activity with support from 2 persons	1	Can stand on the paretic leg up to 5 seconds	1
Can perform the activity with support from 1 person	2	Can stand on the paretic leg for more than 5 seconds	2
Can perform the activity without any help	3	Can stand on the paretic leg for more than 10 seconds	3
**4. Bedside sitting with feet supported on the floor and hands in the lap**		**10. Standing, picking up a shoe from the floor**	
Cannot sit	0	Cannot perform the activity	0
Can sit with slight support, for example with the help of their own hand	1	Can perform the activity with support from 2 persons	1
Can sit for more than 10 s without support	2	Can perform the activity with support from 1 person	2
Can sit for 5 min without support	3	Can perform the activity without any help	3
**5. Sitting to standing up**		**11. Sitting down from standing up**	
Cannot perform the activity	0	Cannot perform the activity	0
Can perform the activity with support from 2 persons	1	Can perform the activity with support from 2 persons	1
Can perform the activity with support from 1 person	2	Can perform the activity with support from 1 person	2
Can perform the activity without any help	3	Can perform the activity without any help	3
**6. Standing with support**		**12. Sitting on edge of bed to supine**	
Cannot stand, even with support	0	Cannot perform the activity	0
Can stand with support from 2 persons	1	Can perform the activity with support from 2 persons	1
Can stand with support from 1 person	2	Can perform the activity with support from 1 person	2
Can stand with only slightly support of 1 hand	3	Can perform the activity without any help	3

UNIVERSITY OF GOTHENBURG

Corrected version 2013-11-29.
Equipment: a stopwatch and a shoe/slipper.
The test is performed with the patient barefoot. Only one attempt per item is allowed. Ensure that the patient meets the criteria for the scores below, as well as the criterion for the registered score. A stopwatch is used in items 4 and 7-9, where the patient should maintain a position within a specific time. In items 1-3, 5-6 and 10-12, the patient´s postural balance/control should be scored according to different degrees of support (verbal, tactile or supervision). The SwePASS, developed by Carina U Persson, Maria Edvinsson, Katharina Stibrant Sunnerhagen and Ulla Svantesson, published in *J Rehab Med* 2011; 43:348-53, is a synthesis of the original French version and the published English version of the Postural Assessment Scale for Stroke Patients by Benaim C, Pérennou DA, Villy J, Rousseaux M, Pelissier JP. *Stroke* 1999; 30: 1862-1868.
Correspondence address: Carina Persson, Institution of Neuroscience and Physiology, Rehabilitation Medicine, Sahlgrenska Academy, Gothenburg University, and Department of Physiotherapy, Sahlgrenska University Hospital/Östra, Gothenburg, Sweden carina.persson@vgregion.se
